# The remodeling roles of lipid metabolism in colorectal cancer cells and immune microenvironment

**DOI:** 10.32604/or.2022.027900

**Published:** 2023-02-03

**Authors:** JIATENG ZHONG, JINGYU GUO, XINYU ZHANG, SHUANG FENG, WENYU DI, YANLING WANG, HUIFANG ZHU

**Affiliations:** 1 Department of Pathology, School of Basic Medical Sciences, Xinxiang Medical University, Xinxiang, 453003, China; 2 Department of Pathology, The First Affiliated Hospital of Xinxiang Medical University, Xinxiang, 453100, China; 3 Department of Anesthesiology, The Third Affiliated Hospital of Sun Yat-sen University, Guangzhou, 510630, China

**Keywords:** Lipid metabolism, Colorectal cancer, Tumor microenvironment (TME)

## Abstract

Lipid is a key component of plasma membrane, which plays an important role in the regulation of various cell biological behaviors, including cell proliferation, growth, differentiation and intracellular signal transduction. Studies have shown that abnormal lipid metabolism is involved in many malignant processes, including colorectal cancer (CRC). Lipid metabolism in CRC cells can be regulated not only by intracellular signals, but also by various components in the tumor microenvironment, including various cells, cytokines, DNA, RNA, and nutrients including lipids. In contrast, abnormal lipid metabolism provides energy and nutrition support for abnormal malignant growth and distal metastasis of CRC cells. In this review, we highlight the remodeling roles of lipid metabolism crosstalk between the CRC cells and the components of tumor microenvironment.

## Introduction

Colorectal cancer (CRC) is the most common malignant tumor of digestive tract in the world. Its incidence rate ranks the third in the incidence rate of all cancers and the second in the death caused by cancer [1,2]. It has been reported that the incidence of CRC is positively correlated with socioeconomic development. By 2030, the number of CRC cases in developed countries is projected to increase to 2.2 million and the number of deaths to 1.1 million [3]. In recent years, although some progress has been made in the early diagnosis and systemic treatment of CRC, the five-year survival rate of patients with CRC is still only about 50%. This is mainly because CRC often has no obvious symptoms in the early stage, and most CRC patients are diagnosed at a late stage and even have metastases. However, the mechanism of the occurrence, development and invasion of CRC is still not completely clear. Therefore, searching for detection methods and tumor markers for early diagnosis of CRC, and further exploring the exact molecular mechanism of its occurrence, development and invasion are hot topics in current research. Epidemiological studies have found that diet, obesity and diabetes are risk factors for CRC [4]. A large number of studies have shown that the abnormal lipid metabolism of cells is involved in the occurrence and development of colorectal cancer, and is significantly related to the clinical treatment effect and prognosis [5].

Lipids are hydrophobic macromolecules, which are divided into several groups according to structure of ketoacyl and isoprene: FAs (fatty acids), phospholipids, TGs (cholesterol, triglycerides), sphingolipids, and cholesteryl esters [6]. Lipids are essential nutrients for cells, acting as the structural components of cell membranes, material transport, energy suppliers, signaling molecules, apoptosis and other aspects [7]. Abnormal lipid metabolism refers to the abnormal anabolism and catabolism of lipids in the body, resulting in too much or too little lipids in each tissue, thus affecting the body function [8]. Although normal cells regulate anabolic and catabolic pathways to adapt to changes in nutrient supply, tumor cells can exhibit uncontrolled proliferation even in the presence of nutrient deficiency. The tumor microenvironment is hypoxic, acidic, and nutrient deficient, resulting in metabolic reprogramming of tumor cells and adjacent stromal cells to promote tumor cell survival, proliferation, and metastasis [9]. Many studies have shown that the abnormal lipid metabolism in cells is significantly related to the induction and metastasis of cancer. At the same time, clinical studies have shown that the abnormal lipid metabolism is closely related to the poor prognosis of CRC patients [10]. Both malignant progression and accelerated proliferation of CRC cells require more energy, which induces changes in lipid metabolism to allow CRC cells survive. Abnormal lipid metabolism causes changes in various genes and proteins, as well as the dysregulation of cytokines and signaling pathways [11]. Attentionally, lipid metabolism in CRC cells can be regulated not only by intracellular signals, but also by various components in the tumor microenvironment, including various cells, cytokines, DNA, RNA, and nutrients including lipids [12].

In this review, we discussed the changes of lipid metabolism in colorectal cancer and its role in the genesis and development of colorectal cancer, and also discussed the remodeling of lipid metabolism pathway in CRC cells and tumor microenvironment. The review provides a summary for better understanding and targeting lipid metabolism therapy and improving prognosis.

## Nutrient Sources for Lipid Metabolism in CRC

**Figure 1:**
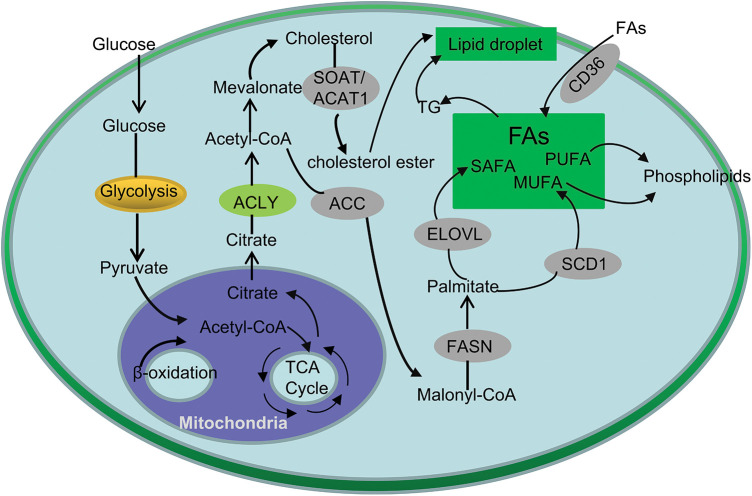
Simplified overview of lipid metabolism in CRC. Glucose can be converted to pyruvate by glycolysis and enter the mitochondria to form citrate. Citrate-derived acetyl-CoA are used in the *de novo* synthesis of cholesterol and FAs. In addition, exogeneous uptake also contributes to the FAs in CRC cells. Cholesteryl esters and TG are stored in lipid droplets.

As the main area of lipid synthesis, glucose is first converted into pyruvate through glycolysis, and further forms citrate in mitochondria, which is released into the cytoplasm of cells as the precursor of FA and cholesterol synthesis [13] (Fig. 1). Studies have shown that the expression of several glucose transporters (GLUT) and related enzymes involved in regulating glycolysis and lipid synthesis in CRC cells is significantly up-regulated. As an important member of the GLUT family, GLUT1 is significantly up-regulated in CRC, and is significantly related to the occurrence, development and prognosis of colorectal cancer [14]. Further research found that GLUT1 gene regulates TGF-β/PI3K AKT mTOR signal affects many biological behaviors of CRC cells [15]. Moreover, GLUT3, a homologous family member of GLUT1, was highly expressed in CRC and negatively linked to CRC patient prognosis [16].

Besides *de novo* synthesis, another major way for cells to obtain FAs is to extract lipids from the external environment (Fig. 1). CD36 transports FAs into cells and plays essential roles in cell growth, metastasis, angiogenesis, immune response, adhesion, and epithelial-mesenchymal transition (EMT) in cancers [17]. For instance, precious study revealed that CD36 inhibits GPC4 ubiquitination via ubiquitin proteasome pathway β-Catenin/c-myc signaling pathway, ultimately down regulates the level of c-myc mediated aerobic glycolysis [18]. In addition, inhibition of FASN (fatty acid synthase) can increase the expression of CD36 in cells, thereby increasing the proliferation of CRC cells, suggesting that the combination of inhibition of CD36 and increase of FASN expression can further increase the anti-cancer effect [19].

## Lipid Metabolism in CRC Cells

Disorder of lipid metabolism is a typical feature of CRC, and its most significant change is increased *de novo* lipid synthesis [20]. First, citrate generates saturated fatty acids (SAFA) under the action of ACLY (ATP-citrate lyase), ACC (acetyl-CoA carboxylase) and FASN. Then, under the action of FADS (fatty acid desaturase), SCD (stearoyl-CoA desaturase) and ELOVL (fatty acid elongase), MUFA (mono-unsaturated fatty acids) and PUFA (polyunsaturated fatty acids) are generated, which generates saturated or mono-unsaturated phospholipids [21] (Fig. 1).

It is well known that phospholipids are one of the main components of cell membranes, which play an key role in the growth and proliferation of cells [22]. It maintains the homeostasis of the cell membrane; on the other hand, it carries out the information transmission of the cell membrane. Phosphatidylcholine (PC) is one of the components of the glycerophospholipid family, which can synthesize other phospholipids such as phosphatidylserine and sphingomyelin, and is one of the main components of cell membranes [23] (Fig.1). Studies have shown that in CRC tissue, the content of PC is significantly increased, which promotes the growth of CRC cells and regulates intracellular signaling pathways [24]. In addition, phospholipase A2 is the key enzyme in the hydrolysis of PC to lysophosphatidylcholine (LPC) [25]. Studies have shown that LPC can activate macrophages, make macrophages stay in M1 type, produce IL-12, IL-1β, TNF-α and IL-6 and other proinflammatory factors, enhancing the inflammatory response, which cause the occurrence of CRC [26,27]. Zhao reported that plasma LPC Levels may represent potential biomarkers for CRC [28].

In the process of FAs synthesis, the key regulatory factors of FA production, including SREBPs (sterol regulatory element binding protein transcription factors), ACLY, ACC, FASN and SCD1, are significantly up-regulated in CRC [29,30]. SREBPs are important transcription factors regulating lipid balance, regulate the synthesis of cholesterol and FAs by promoting the transcription of downstream FASN, ACC, ACLY, SCD1 and other genes [31]. Wen et al. showed that knockdown of SREBPs could significantly inhibit cell proliferation, reduce the proliferation ability of CRC cells, and inhibit the growth of CRC xenografts [32]. FASN is a key downstream factor of SREBP-1 regulated FA *de novo* synthesis, and its expression is significantly elevated in primary CRC and liver metastatic CRCr tissues [33]. On the one hand, FASN can enhance the respiration of cells and maintain the energy homeostasis in cells. It can promote the invasion and metastasis of CRC through Wnt signaling pathway, thereby shortening the survival time of patients [34]. Interestingly, inhibition of SREBPs expression by biotechnology or targeted inhibitors can effectively inhibit tumor growth by inducing CRC cell apoptosis, which makes SREBPs a potential target for tumor targeted therapy [35]. It was found that the expression of ACL Y was significantly up-regulated in CRC, and the down-regulation of gene level and the inhibition of drugs showed significant inhibition of cancer cell growth [36]. HOXA13 (Homeobox A13), a member of the HOX (Homeobox) family, facilitated CRC metastasis by transactivating ACLY. Knockdown of ACLY inhibited HOXA13-medicated CRC metastasis, whereas ectopic overexpression of ACLY rescued the decreased CRC metastasis induced by HOXA13 knockdown [37]. ACC expression is also up-regulated in CRC, and inhibition of ACCs can significantly reduce FA synthesis and inhibit tumor growth in the xenograft model. ACC inhibitors TOFA (5-tetradecyloxy-2-furoic acid), soraphen A, and ND646 showed significant anti-cancer effects in transplanted tumor models [38,39].

FAs are mainly divided into three classes: SAFA, MUFA and PUFA. Due to their different structures, FAs play different roles in the occurrence and development of tumors [40]. Studies have shown that oleicacid, palmitic acid, and linoleicacid can reduce the risk of CRC, while arachidonic acid (AA) and octadecanoic acid significantly increased the risk of CRC [41]. Free AA can produce PGE2, prostaglandin D2 and thromboxane, among which PGE2 is rich in colorectal tumors, which can be up-regulated by β- Catenin, activating PI3K, AKT kinase and RAS mitogen activated protein kinase pathway promote the occurrence of CRC [42].

In addition, newly synthesized FAs are activated under the action of acetyl-CoA synthetase (ACS), and then generate G under the action of GPAT (glycerol-3-phosphate acyltransferase), AGPAT (1-acylglycerol-3-phosphate O-acyltransferase), DGAT (diacylglycerol O-acyltransferase) and PAP (phosphatidic acid phosphatase) [43]. TG is one of the most abundant lipids in human body. Studies have shown that the serum level of TG is positively correlated with the incidence of colorectal adenoma [44]. Excessive TG exists in cells in the form of lipid droplets to provide energy supply and material basis for membrane synthesis. Xiao et al. showed that the accumulation of lipid droplets was significantly increased in human CRC tissues. However, the mechanism between TG and CRC pathogenesis needs to be further studied [45].

At the same time, acetyl-CoA can produce cholesterol through mevalonate pathway, and the accumulation of cholesterol in cells can stimulate macrophages and other immune cells, thus causing inflammatory reaction, At the same time, the content of cholesterol in serum is positively related to the occurrence of CRC [46]. P53 is a common mutant gene in tumors, FreePastor et al. showed that p53 can stimulate the transcription of SREBP, accelerating the synthesis of cholesterol, stimulate the growth of tumor cells [47]. SOAT1 (Cholesterol O-acyltransferase 1), also known as ACAT1 (acyl-CoA acyltransferase 1), converts alcohol into cholesterol ester (Fig. 1). Studies have shown that SOAT 1/is highly expressed in CRC and is inversely proportional to the survival of colorectal cancer patients [48]. Gene knockout of SOAT 1 or the use of its inhibitor can significantly inhibit tumor growth in animal models [49]. In addition, TGs and cholesterol esters in cells are stored in lipid droplets (Fig. 1), which have been observed in many tumors, including CRC. Many studies have confirmed that there are obvious lipid metabolism abnormalities and reprogramming in CRC [50].

## Signaling Pathways Activated by Abnormal Lipid Metabolism in CRC

Recent studies have shown that lipid metabolites can regulate a variety of signaling pathways [51], which are involved in malignant transformation, cell proliferation, migration, EMT and tumor angiogenesis. Oncogenic signaling pathways can also directly or indirectly regulate metabolites and transcription factors involved in lipid metabolism [52].

### PI3K-AKT signaling pathway

Studies have shown that the abnormal activation of PI3K/AKT signaling pathway not only participates in the genesis and development of many tumors, but also plays an important role in tumor lipid metabolism reprogramming [53]. Activation of AKT leads to *de novo* lipid synthesis, which involves two basic processes. One is the shuttle of metabolic intermediates to provide carbon sources for lipid synthesis. Second, AKT can directly or indirectly promote the essential cofactors of lipid metabolism by activating transcription factors or activating related kinases [54,55]. In turn, AKT activation stimulates mammalian target of rapamycin (mTOR), leading to SREBP processing and activation [56]. SREBP induces transcription of target genes, which include many factors involved in lipid metabolism and fatty acid uptake. In addition, this process can promote metabolic reprogramming of glycolysis, which activates *de novo* fat generation [57]. The study by Zhang showed that Hepatocyte growth factor (HGF) affects SREBP dependent cholesterol biosynthesis pathway by regulating c-Met/PI3K/AKT/mTOR axis in CRC cells [58]. The sphingolipid metabolism related proteins LIM and LASP1 (SH3 protein 1) affect mitochondrial membrane sites through PI3K/Akt/mTOR, thereby inducing the occurrence and progression of CRC [59]. Glycogen synthase kinase 3 (GSK3) is the downstream of AKT, and AKT can inhibit the expression of GSK3, thereby promoting the phosphorylation of SREBP and inhibiting the expression of SREBP [60]. Research by Jutao Feng shows that AKT/GSK can trigger the EMT of CRC cells via up regulation of Snail [61]. Insulin plays an important role in lipid synthesis. Insulin can activate the expression of SREBP-1 through the PI3K/AKT/mTORC1/S6K1 signaling pathway, accelerating the *de novo* synthesis of FAs [62]. The study by Liem M showed that insulin can induce continuous cell proliferation by activating PI3K/Akt signaling pathway in cells [63]. In addition, as a member of the PDGF family, VEGF-A can also induce angiogenesis in both physiological and pathological conditions [64]. Research shows that the expression of VEGF-A is significantly related to the prognosis of colorectal cancer patients [65]. Studies have shown that VEGF-A activates PI3K and phosphorylates PIP2 to PIP3 through binding with EGF receptor (EGFR), which is an important second messenger involved in AKT recruitment, activates mTOR, and finally causes cell growth and proliferation [66,67]. PTEN is a negative regulator of PI3K/AKT signal, which is overexpressed in about 60%–70% of colon cancer patients. It regulates lipid synthesis through PI3K-AKT-mTORC-SREBP pathway, making PIP3 dephosphorylated to PIP2 [68]. As a member of PTPs (protein tyrosine phosphatases), PTPRO (protein tyrosine phosphatase receptor type O) has been revealed that PTPRO expression is notably downregulated in CRC liver metastasis compared to the primary cancer, and such a downregulation is associated with poor prognosis of patients with CRC. PTPRO silencing induced the activation of the AKT/mTOR signaling axis, thus promoting *de novo* lipogenesis by enhancing the expression of SREBP1 and its target lipogenic enzyme ACC1 by activating the AKT/mTOR signaling pathway [69].

### PPAR signaling pathway

PPARs (Peroxisome proliferator-activated receptors) are ligand-activated transcription factor belonging to a nuclear hormone receptor superfamily, including PPARα, PPARβ/δ and PPARγ [70]. PPAR, as the main lipid sensor and lipid metabolism regulator, plays a key physiological role [70,71]. PPARα It is mainly involved in fatty acid metabolism, while PPARγ is mainly involved in the regulation of fat production, energy balance and lipid biosynthesis [72,73]. PPARβ/δ is involved in the oxidation of fatty acids in skeletal muscle and myocardium, and it can also participate in the regulation of cholesterol level [74]. Studies have shown that PPAR pathway blocking can induce apoptosis and inhibit the growth of CRC like organs *in vitro* [75].

As a member of PTPs, the role of PTPRO in cell signal transduction has attracted more and more attention. It has been proved that PTPRO attenuation is achieved by suppressing PPAR α and its downstream enzyme, peroxidase acyl coenzyme A oxidase 1 (ACOX1), to reduce FA oxidation rate [69]. High expression PPARβ/δ is closely related to the occurrence and development of colorectal cancer. In this process, arachidonic acid stimulates PPARβ/δ, leads to up regulation of cyclooxygenase (COX)-2 and excessive production of prostaglandin (PG) E2, an activator of colon cancer cells [76].

### AMPK signaling pathway

As a metabolic sensor, AMP activated protein kinase (AMPK) is activated when ATP level in cells is low. Recent studies have confirmed that AMPK can regulate intracellular fatty acid oxidation, lipid synthesis and lipolysis through substrate phosphorylation [77]. AMPK is a heterotrimeric protein, which is composed of three subunits, each of which has multiple phosphorylation sites. AMPK participates in the synthesis and decomposition of lipids by influencing gene transcription and protein phosphorylation, and affects the metabolic process in cells [78]. SREBP1 targeted ACC1, FASN and SCD1 are lipogenic enzymes that promote *de novo* synthesis of cytoplasmic FA [79]. ACC1 catalyzes the carboxylation of acetyl CoA to malonyl CoA, which is the main substrate of FASN. FASN catalyzes the *de novo* synthesis of long-chain FAs in the cytoplasm through the condensation of acyl CoA and malonyl CoA [80]. FASN results in an increase in the synthesis of saturated fatty acids, which are then converted into intracellular monounsaturated fatty acids through SCD1 [81]. The accumulated data showed that ACC was phosphorylated by purified AMPK at three sites: Ser79, Ser219, Ser80, Ser1216, Ser1200 and Ser1215 [82]. Li et al. showed that SREBP1 was directly phosphorylated by AMPK at Ser372, suppressing the proteolytic cleavage of precursor SREBP1 into mature SREBP1, leading to the suppression of hepatic steatosis in diet-induced insulin-resistant mice [83]. TIGAR (TP53-induced glycolysis and apoptosis regulator), which is a downstream target gene of p53, play a prominent role in tumorigenesis at multiple levels [84]. Liu showed that depletion of TIGAR in CRC cells promote lipid peroxidation through decreasing SCD1 expression by AMPK-dependent phosphorylation pathway [85].

The anti-tumor effect of the powerful FASN inhibitor 3664 (TVB-3664) is related to the changes in lipid composition, including the significant reduction of FAs and phospholipids and the increase of lactoceramide and sphingomyelin in xenograft (PDX) from CRC patients sensitive to FASN inhibition, which are regulated by AMPK signaling pathway [86].

Some studies demonstrate that metformin show the potential cytotoxicity on CRC- butyrateresistant (BR) cells, the molecular mechanism of which is taht AMPK phosphorylation is significantly upregulated, whereas the ACC is downregulated, which led to caspase activation and apoptosis [87].

## Abnormal Lipid Metabolism on Immune Microenviroment in CRC

A large number of studies have shown that tumor cells reprogram their metabolic patterns to meet their own proliferation needs in the severely nutrient deficient tumor microenvironment (TME) [88]. This not only includes the Warburg effect, which was first discovered, but also the vigorous reprogramming of lipid metabolism plays an important role in tumorigenesis and development [89]. The changes of lipid metabolism of tumor cells are not only driven by their own needs, but also regulated by other cells, and will also affect the function and metabolism of surrounding cells. TME is divided into non immune microenvironment dominated by tumor cells and fibroblasts (CAF) and immune microenvironment dominated by immune cells [90]. TME contains many types of immune cell subsets, such as CD4^+^ T cells, CD8^+^ T cells, B cells, and dendritic cells (DCs), macrophages, natural killer (NK) cells, etc. Among them, DCs, CD4^+^, and CD8^+^ effector T cells and NK cells are activated to inhibit tumor and prevent immune escape and disease progression [91]. Other immune cells, such as tolerant DCs, immunoregulatory T cell (Treg), tumor associated macrophages (TAMs) inhibits anti-tumor immune response, thereby promoting tumor proliferation, invasion, metastasis and angiogenesis [92]. In conclusion, the communication between CRC cells and their surrounding microenvironment is shaped to promote the growth and progress of CRC cells, and escape immune monitoring through various ways [93].

### Metabolic changes in T cells

Tumor infiltrating T lymphocytes play a key role in tumor immunity effect. T cells are divided into CD4^+^ T cells and CD8^+^ T cells. CD4^+^ T cells are divided into anti-tumor and pro-inflammatory T helper type 1 (Th1) cells, immunosuppression Th2 cells, Th17 cells and Tregs, which secretes immunosuppressive cytokines such as IL-10 and transforming growth factor β (TGF-β), regulating the functions of T cells [94]. The excessive proliferation of tumor cells leads to the lack of nutrition and oxygen in TME, which will reshape the metabolic pathway of T cells from relying on glycolysis for energy supply to relying on FA oxidation and oxidative phosphorylation to maintain the effect function [95]. At the same time, the uptake of lipids increases, and the abnormal accumulation of lipids in cells. However, the accumulated lipid will damage the function of mitochondria in T cells, thus affecting energy supply and immune function. At present, CD4^+^ T cells, CD8^+^ T cells and Tregs are the most frequently studied cell subsets in terms of the effect of lipid metabolism on T cells [96].

In TME, some effector molecules, including interferon γ (IFN-γ) and tumor necrosis factor α (TNF-α), secreted by tumor-infiltrating CD8^+^ T cells, are decreased, while some molecular markers on cancer cells, such as T cell immunoglobulin and mucin domain-containing 3 (TIM-3) and programmed death 1 (PD-1) are increased, indicating that infiltrating CD8^+^ T cells are in a state of exhaustion and cannot play a normal anti-tumor effect [97]. Studies have shown that the increased expression of genes related to FAs metabolism, oxidative stress and ATP production in CRC CD8^+^ TIL of diabetes patients may inhibit the function of CD8^+^ T cells [98]. In addition, the response of CRC CD8^+^ TIL from diabetes patients to cytokine signaling, lipid and glucose is reduced, which in turn will affect its protective function in TME, which is conducive to tumorigenesis and immunosuppression [99]. Acyl-CoA dehydrogenase short-chain (ACADS), a crucial enzyme in the FA metabolism pathway located in mitochondria, expression levels of which were positively related to B cells, CD4^+^ T cells, CD8^+^ T cells, and Tregs in CRC tissues [100]. The research from Song M. showed that Marine ω-3 PUFAs, including eicosapentaenoic acid, docosahexaenoic acid, and docosapentaenoic acid, possess potent immunomodulatory activity and can protect against cancer development [101]. High marine ω-3 PUFA intake was associated with lower risk of CRC with high-level, but not low-level, FOXP3+ T-cell density, suggesting a potential role of ω-3 PUFAs in cancer immunoprevention through modulation of Tregs [102]. In addition, studies showed that a multikinase inhibitor, H89, could promote naïve CD4^+^ T-cell differentiation into Th1, with a decrease in Treg differentiation, and an increase in CD8^+^ T-cell activation and cytotoxicity [103]. In addition, H89 induces overexpression of genes involved in anti-tumor immune response (such as IL-15RA), and its depletion counteracts the anti-tumor effect of H89. At the same time, H89 regulates Akt/PP2A pathway axis and participates in TCR and IL-15 signal transduction. The results show that H89 is a potential strategy for immune system activation, which can prevent and treat CRC [103].

FAs in TME support the proliferation and differentiation of Th17 cells. In order to explore the role of FAs synthesis in Th17 cells, Cluxton et al. added ACC or FASN inhibitor to CD4^+^ T cell culture medium, the proliferation of Th17 cells was inhibited, which proved that Th17 cell proliferation depended on FAs synthesis [104]. In addition, a similar study is that Berod et al. found that the development of Th17 cells depends on ACC mediated *de novo* synthesis of FAs and glycolysis lipogenesis metabolic pathway [105]. Blocking ACC can inhibit the formation of Th17 cells and promote the differentiation of Tregs.

### Metabolic changes in TAMs

TAM is one of the main immune cells in TME, and different TAM subsets can induce or inhibit anti-tumor immunity [106]. TAM is divided into M1 type which inhibits tumor and M2 type which promotes tumor. In TME, the differentiation of macrophages into M1 type depends on aerobic glycolysis [107]. TAM tends to M2 like macrophages, which not only inhibits anti-tumor immune response by regulating the activation and apoptosis of T cells and NK cells, but also promotes tumor cell proliferation, metastasis, angiogenesis and immunosuppression [108]. For example, 1-acylglycero-3-phosphate O-acyltransferase 4 (Agpat 4) silencing induced CRC cells and polarized macrophages to release LPA through LPA receptors 1 and 3 to the M1 like phenotype. This M1 activation is characterized by an increase in p38/p65 signal transduction and proinflammatory cytokines, which promotes the infiltration and activation of CD4^+^ and CD8^+^ T cells in the tumor microenvironment [109]. The accumulation of lipid and the increase of FA oxidation in TAM are necessary for its immunosuppressive activity. In turn, the accumulation of lipid and the increase of FA oxidation will induce TAM to polarize toward M2 phenotype and promote tumor development [110].

In TME, abnormal lipid metabolism regulates the M2 immunosuppressive phenotype of TAM in a variety of ways. First, abhydrolasedomain containing 5 (ABHD5) is a co activator of TG lipase and participates in TG lipolysis [111]. Studies on CRC have shown that TAM contains a large number of lipid droplets and the expression of ABHD5 is up-regulated [112]. ABHD5 inhibits the production of reactive oxygen species in TAM by inhibiting the inflammatory pathway of NLR family containing 3 protein domains (NLRP3), thereby affecting its phagocytosis and killing functions [113]. Therefore, PGE2 is known to play an important role in the polarization of macrophages. The enzymatic degradation of PGE2 involves NAD+dependent 15 hydroxyprostaglandin dehydrogenase (15-PGDH) [114]. Eruslanov et al. reported that the overexpression of 15-PGDH in mouse CRC cells transformed M2 oriented TAMs into M1 oriented macrophages, indicating that PGE2 can induce the phenotype of macrophages to change from anti-tumor M1 macrophages to tumor promoting M2 macrophages [115]. Finally, extracellular vesicles (EVs) are membrane bound vesicles containing different biomolecules and participate in intercellular signal transmission. More and more evidence shows that cancerogenic EVs are absorbed by macrophages and regulate their phenotype and cytokine distribution. Ineta et al. found that EVs derived from CRC cell lines increased CXCL10 and TNF in monocytes-α and IL-23 secretion, and promote the polarization of macrophages [116]. Interestingly, serum CXCL10 and TNF-α Elevated levels are associated with low survival in CRC patients [117]. In addition, Treg and Th17 cells are one of the target cells of IL-23. Therefore, CRC EVs may affect cancer progression through Treg and Th17 cell activation.

### Metabolic changes in DCs

DCs is the main antigen presenting cell, which can process and present antigen polypeptides and express them on major histocompatibility complex (MHC) for T cells to recognize and induce antigen specific immune response [118]. In TME, High fatty acid concentration, up regulation of FASN, activation of TLR and other signals can promote FAs synthesis and lipid accumulation in DCs [119]. First of all, in TME FAs metabolism will affect the development, maturation and function of DCs. Free fatty acid receptor 2 (FFAR2), a receptor for short-chain FAs has been showed that loss of FFAR2 promotes colon tumorigenesis in mice by promoting exhaustion of CD8^+^ T cells, and overactivating DCs, leading to their death. Additionally, the lipid accumulated in the DCs will also lead to the obstacle of antigen cross expression [120]. Tumor infiltrating DCs usually accumulates in the liposome, which can covalently bind with heat shock protein 70 (p70) to prevent peptide MHC complex from transporting to the cell surface, and then lead to the accumulation of peptide MHC complex in the late endosome/lysosome [121]. O’Toole et al. found that CRC patient DCs secreted low levels of IL-12p70 and failed to upregulate expression of maturation markers in response to LPS, suggesting that lipid accumulation in DCs can reduce antigen processing capacity and weaken the ability to stimulate T cell response, leading to DCs dysfunction [122].

### Metabolic changes in cancer-associated fibroblasts (CAFs)

Cancer associated fibroblast (CAF) is one of the main components of stromal cells around cancer cells. Recent studies have shown that CAF secretes chemokines, growth factors, extracellular matrix and matrix metalloproteinases to regulate the occurrence, development and metastasis of tumors [123]. In addition, recent studies have shown that the metabolic interaction between CAF and tumor cells affects tumor metastasis [89]. First of all, in TME, CAF needs metabolic reprogramming to adapt to the severe lack of nutrition and oxygen, and the increased demand for energy necessary to maintain the high proliferation of CRC. At present, research on lipid metabolism reprogramming of CAFs mainly focuses on *de novo* synthesis and catabolism of FAs [123,124]. Gong et al. reported that compared with normal fibroblasts, FAs, diglycerides (DGs), phosphatidic acid (PA), phosphatidylinositol (PI), LPC and phosphatidylethanolamine (PE) were significantly up-regulated in CAFs, accompanied by higher levels of FAs and phospholipids excretion [123]. Metabolic reprogramming of CAF leads to increased FASN expression, while increased FAs uptake in CRC cells leads to metastasis. Peng Shaoyong et al. used lipomics to reveal that CAF increases the fluidity of cell membrane by up regulating the unsaturated acyl chain in the PC in cells, thereby increasing the migration of CRC cells. In addition, using non targeted metabonomics, it has been found that CRC cells can absorb lipid/lipid metabolites from CAF to compensate for the low expression of SCD [125]. In addition, CRC cells can also secrete FA metabolites to affect the migration function of CAF. For example, CRC cell-derived 12 (S)—HETE, an proinflammatory AA metabolite, triggers a signal that is transduced by PLC, IP3, free intracellular Ca^2+^, Ca^2+^-calmodulin kinase II, RHO/ROCK and MYLK, leading to the activation of myosin light chain 2, and the subsequent mobility of CAF [126].

## Conclusion

CRC is a complex disease with multiple genes, multiple steps and multiple stages, which is marked by genetic changes, signal pathway disorders and metabolic remodeling. Although the mortality rate of CRC has declined in the past 10 years, it still threatens human health, especially with the increase of incidence rate of young patients. The disorder of lipid metabolism has become a recognized feature of several cancers, mainly due to advances in technology in this field. Specifically, high-throughput methods such as lipomics, chemical imaging and functional genomics make lipid identification and characterization possible. Some studies use this method to describe the lipid metabolism characteristics of various cancers, including CRC [127].

**Figure 2:**
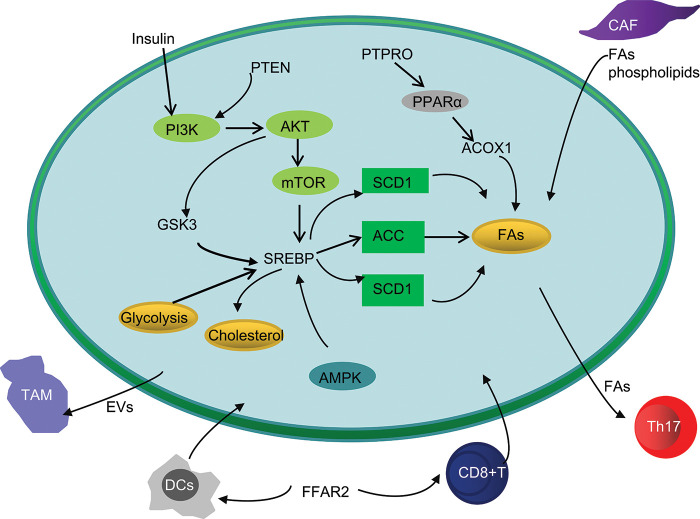
Simplified overview of abnormal lipid metabolism interaction between CRC cells and TME.

Abbreviations: GSK3, Glycogen synthase kinase 3; PTEN, Phosphatase and tensin homolog; PTPRO, protein tyrosine phosphatase receptor type O; PPAR, Peroxisome proliferator-activated receptor; ACOX1, acyl-coenzyme A oxidase 1; AMPK, AMP-activated protein kinase; CAF, cancer-associated fibroblast; DCs, dendritic cells; TAMs, tumor associated macrophages; FFAR2, Free fatty acid receptor 2.

In this review, we summarize the characteristics of abnormal lipid metabolism in CRC and describe the signaling pathways activated by abnormal lipid metabolism that lead to the occurrence, development, and metastasis of CRC. In addition, abnormal lipid metabolic interactions between cancer cells and Tmes during CRC progression are discussed. Remodeling of lipid metabolism of immune cells or competition with cancer cells for FAs can lead to tumor immunosuppression and immune escape. At the same time, other components of TME, such as CAF, can also support the metabolic needs of CRC cells by secreting FAs, thus leading to tumor invasion and metastasis (Fig. 2).

A growing number of studies have confirmed that lipid metabolism has been involved in various metabolic pathways of CRC cell biology [128]. The biosynthesis, uptake and modification of lipid not only affect the proliferation and survival of CRC cells, but also affect tumor cell migration, invasion and tumor angiogenesis through more complex signal pathways and remodeling of TME. At present, some inhibitors of FA metabolizing enzymes and transcription factors have started preclinical and clinical anti-tumor treatment research [129]. The screening of natural active compounds targeting tumor lipid abnormal metabolic pathways and related enzymes and the development of new drugs will also open up new areas for the treatment of CRC.

## Abbreviations

FAs: Fatty acids
ACLY: ATP-citrate lyase
ACC: Acetyl-CoA carboxylase
FASN: Fatty acid synthase
CD36: Fatty acid translocase/scavenging receptor
ELOVL: Fatty acid elongase
SCD1: Stearoyl-CoA desaturase 1
TG: Triacylglycerol
SOAT1: Sterol O-acyltransferase
ACAT1: Acyl-CoA acyltransferase 1
SAFA: Saturated fatty acids
PUFA: Polyunsaturated fatty acids
MUFA: Mono-unsaturated fatty acids
